# Mental health problems in unaccompanied young refugees and the impact of post-flight factors on PTSS, depression and anxiety–A secondary analysis of the Better Care study

**DOI:** 10.3389/fpsyg.2023.1149634

**Published:** 2023-06-20

**Authors:** Fabienne Hornfeck, Jenny Eglinsky, Maike Garbade, Rita Rosner, Heinz Kindler, Elisa Pfeiffer, Cedric Sachser

**Affiliations:** ^1^German Youth Institute, Munich, Germany; ^2^Department of Child and Adolescent Psychiatry/Psychotherapy, Ulm University, Ulm, Germany; ^3^Department of Psychology, Catholic University Eichstätt-Ingolstadt, Eichstätt, Germany

**Keywords:** unaccompanied young refugees, trauma, daily stressors, PTSS, depression, anxiety, mental health, child welfare services

## Abstract

**Background:**

Unaccompanied young refugees (UYRs) show elevated levels of mental distress such as post-traumatic stress symptoms (PTSS), depression, and anxiety. The individual post-arrival situation in the host country plays an important role in increasing or reducing mental health risks for these vulnerable children and youth. The study aims at examining the impact of pre- and post-migration factors on the mental health of UYRs.

**Methods:**

A cross-sectional survey of *N* = 131 young refugees (81.7% male, *M* = 16.9 years old) was conducted in 22 children and youth welfare service (CYWS) facilities in Germany. The participants provided information about pre- and post-flight experiences. Standardized measures were used to assess post-traumatic stress symptoms (CATS-2), symptoms of depression (PHQ-9), and anxiety (GAD-7). Daily stressors were assessed with the Daily Stressors Scale for Young Refugees (DSSYR), sociocultural adaptation with the Brief Sociocultural Adaptation Scale (BSAS), satisfaction with social support with the Social Support Questionnaire (SSQ6-G).

**Results:**

Our results demonstrated clinical levels of PTSS in 42.0% of the participants, depression in 29.0%, and anxiety in 21.4%. Hierarchical regression analyses revealed that a higher number of traumatic events and social daily stressors predicted higher levels in all three domains of mental health problems. PTSS and anxiety were also predicted by the distress related to the residence status, depressive symptoms were additionally predicted by sociocultural adaptation, less family contact and length of stay. The satisfaction with social support was not a significant predictor in the regression models.

**Conclusion:**

Unaccompanied young refugees in CYWS facilities are a highly vulnerable population. As traumatic events, daily stressors and level of contact to family directly impacted UYRs mental health, interventions should be trauma-focused, but also contain modules on how to cope with daily stressors. On the policy and practical level, stakeholders in host countries are called for establishing measures to reduce post-migration stressors and enhance support for UYRs on all levels.

## 1. Introduction

Among the refugee population, unaccompanied young refugees (UYRs) are often considered as the most vulnerable group–due to their young age and the lack of protection from a primary caregiver (Bean et al., 2007; Derluyn and Broekaert, 2008; Derluyn and Vervliet, 2012; Ingleby et al., 2012). Due to their precarious situation, UYRs have a special need for protection (UNHCR, 1997). It is well documented that UYRs are especially vulnerable to mental distress such as post-traumatic stress disorder (PTSD), depression, and anxiety (Fazel et al., 2012; Barghadouch et al., 2018; Norredam et al., 2018). Findings on the prevalence of psychological disorders based on screening questionnaires or clinical interviews are extremely heterogeneous. In a systematic review, El Baba and Colucci (2018) reported a high prevalence of post-traumatic stress symptoms (19.5%–67.3%), depressive symptoms (14.6–35.1%) and anxiety (25–36%) in UYRs arriving and living in a host country. Studies have also documented that these problems persist over time even several years after the flight experience and resettlement in the host country (Seglem et al., 2011; Eide and Hjern, 2013; Jensen et al., 2014; Jakobsen et al., 2017; Müller et al., 2019).

However, many UYRs show a remarkable resilience and high levels of functioning in their every-day lives, despite the experience of potentially traumatic events (Keles et al., 2018a; Popham et al., 2023). Hence, a growing number of researchers in the field attempt to identify factors that predict mental health outcomes of young refugees.

A considerable amount of literature has analyzed the role of risk factors affecting the mental health of young refugees before and during flight (e.g., gender, flight duration, country of origin). Several studies have documented that UYRs have experienced a wide range of adverse events before and during their flight (Bean et al., 2007; Fazel et al., 2012; Jensen et al., 2015). In a systematic review, Höhne et al. (2020) found that the number of stressful life events (e.g., exposure to war traumata or experience of violence) was the most important risk factor for UYRs mental health, indicating that a greater number of stressful life events is associated with more mental health problems. The number and severity of stressful life events has also been confirmed as risk factor for PTSS, depressive symptoms, and anxiety in a systematic review of El Baba and Colucci (2018). However, traumatic experiences do not necessarily lead to enduring mental health problems (Hodes et al., 2008; Carlson et al., 2012; Keles et al., 2018a), hence psychosocial factors and the interplay of individual and external stressors seem to play an important role in predicting young refugees’ distress (Fazel et al., 2012; Höhne et al., 2020). Therefore, the identification of post-migration risk and protective factors for UYRs mental health is crucial for enabling a healthy development.

### 1.1. Post-migration factors

After arrival in the host country, refugees usually continue to face many challenges. Findings suggest that the impact of war and forced migration on mental health is compounded or alleviated by the post-migration resettlement context (Fazel et al., 2012; Höhne et al., 2020). Upon arrival in the host country, young refugees must still cope with the traumatic memories of past experiences and additionally might face stressors in the host country (like e.g., learn a new language, adapt to the new culture, deal with uncertainties about the future, worry about the family, loneliness etc.), which are summarized as post-migration stressors. In this new and uncertain life situation, the separation from their family members puts UYRs additionally at risk (Fazel et al., 2012; El Baba and Colucci, 2018). There are different models explaining the underlying processes of associations between post-migration stressors and mental distress. According to an ecological model of refugee distress proposed by Miller and Rasmussen (2017), ongoing stressors may prevent recovery from losses and traumatic experiences of war and might even hinder the effectiveness of therapeutic interventions through the depletion of functional coping mechanisms. In contrast, the “stress sensitivity theory” (Gunnar and Quevedo, 2007) proposes that traumatic experiences lead to an overreaction to ongoing demands and consequently decrease the tolerance for stressors. Additionally, the post-flight stressors themselves and their accumulation and continuance could increase the risk of serious mental health problems, like e.g., anxiety or depression (Bronstein and Montgomery, 2011). Hence, post-migration stressors seem to have a direct effect on mental distress, as well as indirect effects by preventing recovery and exacerbating symptoms from trauma.

#### 1.1.1. Daily stressors

To date, there are only a few studies that shed light on the effect of daily stressors in UYRs, like e.g., perceived discrimination, stress related to legal procedures, living circumstances, economic concerns, worries about family members, and difficulties making friends (Seglem et al., 2011; Vervliet et al., 2014; Keles et al., 2018b; Jensen et al., 2019; Dangmann et al., 2021). All of these studies were conducted in Western European countries and found that higher levels of daily stressors were associated with more mental health problems. Furthermore, a number of studies have focused on the differential impact of stressors like e.g., the impact of accommodation placement on psychological wellbeing (O’Higgins et al., 2018). Recent evidence indicates that being placed in a low support facility with greater restrictions presents a risk factor for the mental health of young refugees (Geltman et al., 2005; Jakobsen et al., 2017; Mitra and Hodes, 2019; Höhne et al., 2020). Accommodation in restrictive reception centers or detention centers negatively affects the wellbeing of UYRs, especially in terms of emotional problems (Reijneveld et al., 2005; Ehntholt et al., 2018), whereas higher care levels turned out to be beneficial for UYRs mental health (Bean et al., 2007; Hodes et al., 2008). Another factor that influences mental health and behavioral outcomes is the practice of multiple relocations within the asylum system (Nielsen et al., 2008). A review on studies investigating the impact of factors associated with the asylum process on mental health concluded, that refusal of asylum and insecure status had a negative effect on the wellbeing of UYRs as it produces a situation of instability and fear of return (Hornfeck et al., 2022). In another quantitative study, the fear of deportation was associated with mental distress (Höhne et al., 2021). However, a systematic understanding of what exactly is stressing UYRs during asylum procedures is still lacking.

#### 1.1.2. Social support

There are several studies with refugee children and adolescents which highlight the protective effect of social support after resettlement for mental health and its importance for recovering after trauma (Fazel et al., 2012; Verelst et al., 2022). A study with UYRs in Germany by Sierau et al. (2018) showed that support from different social networks may have different effects on youth’s mental health trajectories. Especially support from an adult mentor moderated the association between the number of stressful life events the youth had experienced and symptoms of PTSD, depression, and anxiety. A moderating effect of social support on the relationship between acculturative stress and anxious-depressed symptoms was found in a study with accompanied immigrant adolescents (Sirin et al., 2013). Oppedal and Idsoe (2015) found direct effects of social support on depression, but not PTSD symptoms in a group of resettled UYRs. Social support also predicted mental health problems in a recent study of Syrian refugee children from Popham et al. (2023).

#### 1.1.3. Family contact

In prior studies, more contact with family members was found to be associated with better mental health outcomes in UYRs (Hollins et al., 2007; Oppedal and Idsoe, 2015; Sierau et al., 2019; Höhne et al., 2021; Behrendt et al., 2022). However, in another study with UYRs, no effect of family support was found (Sierau et al., 2018).

#### 1.1.4. Sociocultural adaptation

Adapting to a new culture and environment also implies more practical and behavioral aspects, like adapting to an unknown school system, learning a new language, getting along in an unknown climate zone etc., Montgomery and Foldspang (2007) found associations between social adaptation and internalizing and externalizing behavior in young refugees. Evidence has also been presented that school attendance and language skills can have a protective effect on mental health of UYRs (Höhne et al., 2021).

#### 1.1.5. Duration since arrival

Analyses on the association between length of stay in the host country and the level of mental health problems do not yield a consistent picture (Scharpf et al., 2021). Therefore, the time spent in a host country as an indicator may be entangled with the circumstances of the living situation and might only lead to better mental health outcomes in a favorable and stable environment.

### 1.2. Study aims and research questions

Although the number of studies on post-migration stressors has increased in recent years, there is still a great need for studies using validated instruments that investigate the differential impact of factors and controlling for the cumulative effect of traumatic events on the mental health of resettled UYRs.

This study aims to assess the current level of mental health problems in UYRs 5 years after the increased movement of refugees to European countries. Based on the literature, we expect high levels of PTSS, depression and anxiety in our sample of UYRs. The study further aims to expand the evidence base for the contribution of post-migration factors and sociocultural aspects to the mental health of UYRs. We suppose that UYRs reporting more post-migration stressors, less family contact, low satisfaction with social support, more distress regarding the asylum status, and a poorer adaptation to the new culture show poorer mental health outcomes with regard to PTSS, depression, and anxiety.

## 2. Materials and methods

The study was approved by the ethics review board of the University Ulm (243/19) and Eichstätt-Ingolstadt (004-19). The BETTER CARE study was registered in German Clinical Trials Register^1^ and a study protocol was published (Rosner et al., 2020).

### 2.1. Procedure

The data used in this study were derived from the above-mentioned trial and baseline data of a predefined subsample were analyzed. The study was conducted in residential group homes for UYRs in four German federal states. After agreement with children and youth welfare services (CYWS) facilities, screenings with UYRs were organized with the help of social workers and caregivers in the facilities. If needed, interpreters were available in person. Prior to assessment, UYRs were fully informed about objectives, procedure and content of the study. Inclusion criteria for participants were (1) age 12−20 years, (2) arrived in Germany as unaccompanied minors, (3) applied for asylum or intend to do so, (4) living in a CYWS facility, (5) written informed consent given by participant and legal guardian (if <16 years at screening), and (6) reported at least one traumatic event in line with the DSM-5 A criterion.

### 2.2. Sample

Recruitment and screenings of UYRs took place between July 2020 and July 2021 in 22 CYWS facilities. The final sample consisted of *N* = 131 UYRs. 81.7% of the participants were male and 1% indicated a diverse gender. The age at assessment ranged between 13 and 20 years (*M* = 16.95; SD = 1.46) and they had resided between 1 and 90 months in Germany (*M* = 25.75; SD = 20.52). The participants came from 29 different countries of origin, most participants (30%) were born in Afghanistan. Demographic information on the participants is presented in Table 1.

**TABLE 1:** Sociodemographic characteristics of participating UYRs.

	*M* (*SD*); range	*n* (%)
Age 16.95 (1.46); 13−20		
**Gender**		
Male		107 (81.7)
Female		23 (17.6)
Diverse		1 (0.8)
Current school attendance 115 (87.8)		
**Country of origin**		
Afghanistan		40 (30.5)
West Africa^a^		23 (17.7)
East Africa^b^		21 (16.0)
Syria		12 (9.2)
Iran		8 (6.1)
**Religion**		
Muslim		107 (84.9)
Christian		15 (11.9)
Buddismen		1 (0.8)
Other		3 (2.4)
**Residence status**		
Temporary residence permit		43 (32.8)
Pending process		48 (36.6)
Negative decision, tolerated stay		21 (16.0)
Negative decision		3 (2.3)
Other		16 (12.2)

M, mean; SD, standard deviation. ^a^ West Africa: Guinea, Sierra Leone, Gambia, Mali, Nigeria, Benin, Ivory Coast. ^b^ East Africa: Somalia, Ethiopia, Eritrea, Kenia.

### 2.3. Measures

All questionnaires were available in German, English, French, Arabic, Dari, Farsi, Pashtu, Somali, Tigrinya, Russian, and Kurmanci. Assessment of demographic information included age, education and information concerning residential status, the current living situation, and family contact. Two items on distress and anxiety regarding the current asylum status were rated on an 11-point Likert scale from 0 to 10. Contact with family members was rated from 0 (*no contact*) to 5 (*daily contact*).

The *Child and Adolescent Trauma Screen* (CATS-2: Sachser et al., 2022) was used to assess PTSS according to DSM-5 and ICD-11 criteria in children and adolescents. First, individual histories of trauma are examined by a traumatic event checklist consisting of 15 items. The severity of post-traumatic stress symptoms is measured via 25 items and responses on a 4-point scale ranging from 0 (*never*) to 3 (*almost always*). In the current study, we used the DSM-5 total symptom score ranging from 0 to 60 and a cut-off of 25 was set indicating a clinically relevant PTSS. Internal consistency (Cronbach’s α = 0.92) in our sample was found to be excellent.

The *Patient Health Questionnaire* (PHQ-9: Kroenke et al., 2001; Kroenke and Spitzer, 2002) was used to measure symptoms of depression in our study. The items are rated on a 4-point scale ranging from 0 (*not at all*) to 3 (*nearly every day*) with a total score of 0 to 27 indicating the degree of impairment over the past 2 weeks. Based on the validation study by Kroenke et al. (2001), scores of 10 and higher are classified as clinically relevant. The PHQ-9 has been validated in many contexts and languages (Kroenke et al., 2001, 2010) and showed good reliability (Cronbach’s α = 0.83) in the current sample.

The *Generalized Anxiety Disorder Scale* (GAD-7: Spitzer et al., 2006) is a 7-item rating scale based on diagnostic criteria of DSM-4 for generalized anxiety disorder and is widely used in research and clinical practice. The items are rated on a 4-point scale ranging from 0 (*not at all*) to 3 (*nearly every day*) with a total score of 0 to 21 indicating the degree of impairment over the past 2 weeks. Scores of 10 or more indicate the presence of clinically relevant levels of anxiety. The GAD-7 has been validated in many contexts and languages (Kroenke et al., 2010). In our sample a good reliability (Cronbach’s α = 0.85) was indicated.

The *Brief Sociocultural Adaptation Scale* (BSAS: Demes and Geeraert, 2013) is a 12-item questionnaire assessing various aspects of sociocultural adaptation in everyday life (e.g., language, climate, people, values and beliefs). The participants were asked to rate the 12 items on a 7-point Likert-type scale from 1 (*very difficult*) to 7 (*very easy*) and an overall sum score between 12 and 84 was calculated. The scale demonstrated acceptable reliability (Cronbach’s α = 0.79) and is available and validated in different languages. In 2 cases of the current sample (1.5%), data on this scale was missing.

The questions on social support are based on the *Social Support Questionnaire* (SSQ6-G: Leppin et al., 1986). Solely the item assessing satisfaction and the need for more or less support was included in the analysis. 62.5% are “satisfied as it is,” 16.7% “would prefer to have more support,” 15.0% “wish that people would have more time for me, when I’m addressing myself to them“, and 5.8% “would like to have fewer social support.” Due to the skewed distribution of the data, a dichotomous variable was calculated for correlation and regression analyses with 0 indicating dissatisfaction with social support and 1 indicating satisfaction with social support. Information was missing for one case (0.7%).

The *Daily Stressors Scale for Young Refugees* (DSSYR: Vervliet et al., 2014) assesses to what extent 19 different post-migration daily stressors were experienced by the participants during the last month. Based on Behrendt et al. (2022), seven items were clustered indicating the amount of social stressors (“difficulties in making new friends,” “difficulties to communicate with others due to the foreign language,” “feeling bored,” “feeling uncertain about the future,” “hear people say bad things about me,” “feeling of being treated unfairly compared to others,” and “feeling that others have prejudices about me or people of my country/culture”), and nine items form the factor material stressors (“not enough food,” “not enough clothing,” “not enough money,” “not enough housing,” “not enough medical care,” “not enough education,” “lack of information,” “feelings of unsafety,” and “being forcibly and repeatedly moved”). The remaining three items were dropped. A sum score and a mean score of each factor was calculated. In 2 cases (1.5%), the mean scores could not be calculated due to too many missings. The questionnaire is available in many languages and showed good reliability (Cronbach’s α = 0.86) in our study.

### 2.4. Statistical analyses

Analysis was performed using IBM SPSS statistics version 28. Associations among continuous variables were calculated using bivariate, point-biserial correlations [Pearson correlation coefficients (r) reported]. In the case of skewed data, we conducted an additional Spearman correlation analysis. Three separate hierarchical multiple regression analyses were conducted to investigate the impact of pre- and post-flight factors on UYRs’ PTSS, depression and anxiety. In a first step of the model, sociodemographic factors (age and gender) were included as control variables, the number of traumatic events were added in a second step, and post-migration factors (distress regarding residential status, length of stay, family contact, sociocultural adaptation, material and social stressors, and satisfaction with social support) were included in a third step. Due to the high intercorrelation with the variable “worries about deportation,” only “distress regarding residential status” was included. All tests were two-tailed, and an alpha level of *p* < 0.05 was used.

## 3. Results

### 3.1. Preliminary analyses

Table 2 presents descriptive data for the CATS-2, PHQ-9, GAD-7, and pre- and post-migration factors.

**TABLE 2:** Descriptive data on mental health outcomes and pre- and post-migration stressors of participants.

	*M* (*SD*); range	*n* (%)
CATS-2 sum score	24.56 (11.45)	
PHQ-9 sum score	8.69 (5.55)	
GAD-7 sum score	7.10 (4.80)	
Number of PTEs	6.56 (3.05); 1−14	
Frequency of UYRs reporting satisfaction with social support		75 (57.7)
Sociocultural adaptation	4.90 (0.91); 2−7	
Number of social stressors	4.09 (1.83); 0−7	
Number of material stressors	3.69 (2.46); 0−9	
Distress regarding residential status	5.60 (3.48)	
Worries about deportation	5.83 (4.14)	
Family contact	2.27 (1.96)	
No contact		46 (35.1)
Once a year or less		9 (6.9)
Several times a year		9 (6.9)
Monthly		16 (12.2)
Weekly		32 (24.4)
Daily		19 (14.5)

M, mean; SD, standard deviation; PTE, potential traumatic event; CATS, child and adolescent trauma screen; PHQ, patient health questionnaire; GAD, generalized anxiety disorder scale.

Analyses of the frequencies revealed that 48.1% of UYRs showed clinically relevant levels of PTSS, 42.0% scored over the clinical cut-off for depressive symptoms and 29.0% for anxiety. 21.4% showed elevated levels in all three domains and 41.2% showed temporal resilience ranging below the cut-offs in all areas.

Bivariate correlations between all included variables were examined (Table 3). The results indicated that higher PTSS scores were associated with more distress regarding residential status, with a higher number of experienced traumatic events and more daily stressors. Additionally, less family contact, sociocultural adaptation and satisfaction with social support were also associated with higher PTSS scores. The same results were also found for higher anxiety and depression scores (with one exception – higher depression scores seem to be associated with less distress regarding residential status).

**TABLE 3:** Bivariate correlations between included variables.

	n	1	2	3	4	5	6	7	8	9	10	11	12	13
1. Age	127	-												
2. Gender	127	0.060	-											
3. Length of stay	127	0.297***	-0.039	-										
4. Distress regarding residential status	127	0.97	-0.006	–182*	-									
5. Family contact	127	-0.017	-0.171	0.303***	-0.181*	-								
6. Number of PTEs	127	0.059	0.067	0.008	0.366***	-0.203*	-							
7. PTSS	127	0.085	0.085	-0.104	0.467***	-0.278**	0.600***	-						
8. Depression	127	0.047	0.156	0.056	-0.262**	-0.237**	0.419***	0.738***	-					
9. Anxiety	127	0.010	0.084	-0.004	0.355***	-0.303***	0.430***	0.785***	0.813***	-				
10. Sociocultural adaptation	127	-0.091	-0.270**	0.092	-0.211*	0.115	-0.251**	-0.357***	-0.451***	-0.319***	-			
11. Social stressors	127	0.154	0.123	-0.023	0.150	-0.153	0.283**	0.458***	0.497***	0.412***	0.373***	-		
12. Material stressors	127	-0.079	-0.050	-0.172	0.194*	-0.040	0.243**	0.333***	0.452***	0.293**	-0.291**	0.420***	-	
13. Satisfaction with social support	127	0.041	-0.038	0.034	-0.257**	0.160	-0.284***	-0.352***	-0.211*	-0.231**	0.353***	-0.229**	–0.248**	-

*p < 0.05, **p < 0.01, and ***p < 0.001. PTE, potential traumatic event; PTSS, post-traumatic stress symptoms.

To control for effects of the origin on mental health outcomes, three separate ANOVAs with mental health outcomes as dependent variables and region of origin as independent variable were conducted. Four categories for the region were built: Middle East and North Africa, West Africa, East Africa, and others. No effect of the origin on the level of PTSS, depression, or anxiety was found.

### 3.2. Prediction of mental health outcomes

Tables 4–6 present the results of the three stepwise regression models. A significant regression equation was found for the second [*F*(3,122) = 23.20, *p* < 0.001] and third step [*F*(10,115) = 13.47, *p* < 0.001] of the hierarchical linear regression with PTSS as dependent variable. The whole model explained a significant proportion of variance (*R*^2^ = 0.540, *R^2^_*Adjusted*_* = 0.499). The number of traumatic events, distress regarding residential status and social stressors turned out to be significant predictors of PTSS. In the hierarchical multiple linear regression model for symptoms of depression, the predictors included in step 2 [*F*(3,122) = 10.41, *p* < 0.001] and step 3 [*F*(10,115) = 11.13, *p* < 0.001] explained a significant amount of variance (*R*^2^ = 0.492, *R^2^_*Adjusted*_* = 0.448). Depression was significantly predicted by the number of traumatic events, material and social stressors, the time spend in Germany, contact frequency with family members, and sociocultural adaptation. Finally, in the hierarchical regression analysis of symptoms of anxiety as dependent variable [*F*(10,115) = 7.38, *p* < 0.001, *R^2^* = 0.391, *R^2^_*Adjusted*_* = 0.338], the results showed that the number of traumatic events, social stressors, distress regarding the residential status, and contact frequency with family members significantly predicted anxiety scores. Traumatic experiences explained 18.9% of the variance, and post-migration factors explained 19.5% of the variance. Overall, the most important predictors in all three models were the number of traumatic events and social stressors.

**TABLE 4:** Hierarchical regression with post-traumatic stress symptoms (CATS-2) as dependent variable.

Variable	B	95% CI for B		SE B	β	Δ *R*^2^
		*LL*	*UL*			
Step 1 Constant, gender, age						0.015
Step 2 Constant, gender, age, number of PTEs						0.349***
Step 3						0.176***
Constant	10.07	−11.65	31.78	10.96		
Age	0.07	−1.02	1.15	0.55	0.01	
Gender	−0.17	−3.94	3.59	1.89	−0.01	
Number of PTEs	1.42***	0.88	1.97	0.27	0.38	
Length of stay	−0.01	−0.09	0.07	0.04	−0.02	
Distress regarding residential status	0.75**	0.28	1.21	0.24	0.23	
Family contact	−0.58	−1.41	0.26	0.42	−0.10	
Sociocultural adaptation	−0.93	−2.84	0.99	0.97	−0.07	
Social stressors	10.22**	3.60	16.83	3.34	0.23	
Material stressors	2.24	−4.01	8.50	3.16	0.05	
Satisfaction with social support	−1.87	−5.19	1.44	1.67	−0.08	
*R^2^* = *0.540*						

N = 126, **p < 0.01, and ***p < 0.001. PTE, potential traumatic event.

**TABLE 5:** Hierarchical regression with symptoms of depression (PHQ-9) as dependent variable.

Variable	B	95% CI for B		SE B	β	Δ R^2^
		*LL*	*UL*			
Step 1 Constant, gender, age						0.024
Step 2 Constant, gender, age, number of PTEs						0.179***
Step 3						0.288***
Constant	13.86	2.89	24.84	5.54		
Age	−0.38	−0.93	0.17	0.28	−0.10	
Gender	0.47	−1.43	2.37	0.96	0.04	
Number of PTEs	0.35*	0.07	0.62	0.14	0.19	
Length of stay	0.06**	0.02	0.10	0.02	0.22	
Distress regarding residential status	0.17	−0.07	0.41	0.12	0.11	
Family contact	−0.52**	−0.94	−0.10	0.21	−0.18	
Sociocultural adaptation	−1.61**	−2.58	−0.65	0.49	−0.26	
Social stressors	5.10**	1.76	8.44	1.69	0.24	
Material stressors	4.57**	1.41	7.73	1.60	0.23	
Satisfaction with social support	0.97	−0.70	2.65	0.84	0.09	
*R^2^* = *0.492*						

N = 126, *p < 0.05, **p < 0.01, and ***p < 0.001. PTE, potential traumatic event.

**TABLE 6:** Hierarchical regression with symptoms of anxiety (GAD-7) as dependent variable.

Variable	B	95% CI for B		SE B	β	Δ *R*^2^
		*LL*	*UL*			
Step 1 Constant, gender, age						0.007
Step 2 Constant, gender, age, number of PTEs						0.189***
Step 3						0.195***
Constant	10.98	0.51	21.45	5.29		
Age	−0.42	−0.94	0.11	0.26	−0.13	
Gender	−0.23	−2.04	1.58	0.92	−0.02	
Number of PTEs	0.34*	0.07	0.60	0.13	0.21	
Length of stay	0.04	−0.00	0.08	0.02	0.16	
Distress regarding residential status	0.30**	0.08	0.53	0.11	0.22	
Family contact	−0.55**	−0.96	−0.15	0.20	−0.22	
Sociocultural adaptation	−0.73	−1.65	0.20	0.47	−0.14	
Social stressors	4.42**	1.23	7.61	1.61	0.24	
Material stressors	1.06	−1.95	4.08	1.52	0.06	
Satisfaction with social support	0.27	−1.33	1.86	0.81	0.03	
*R^2^* = *0.391*						

N = 127, *p < 0.05, **p < 0.01, and ***p < 0.001. PTE, potential traumatic event.

## 4. Discussion

During the past two decades, there has been a growing interest in the investigation of post-migration stressors and their impact on the mental health of young refugees (Fazel et al., 2012; Höhne et al., 2020). The present study extends the knowledge on prevalences of mental health problems in UYRs living in the unique setting of residential care facilities and adds further insight into the role of post-migration factors. In the present sample, the frequencies of clinically relevant levels of PTSS (48.1%), depressive symptoms (42%), and anxiety (29%) fell within the wide range of prevalences reported in previous studies on young refugees (e.g., Witt et al., 2015; El Baba and Colucci, 2018) and emphasize the high vulnerability of UYRs. Compared to a German study with UYRs in group homes (Sierau et al., 2019), the authors reported similar frequencies of clinically relevant levels of depression (42.0%) and anxiety (23.8%), but lower prevalences for PTSS (32%) than in the present study. However, there is a substantial number of UYRs in the present study (41.2%) with no identifiable mental health problems despite adverse experiences before, during and after flight. This is in line with more recent research and practice highlighting the resilience of young refugees and a growing number of studies attempting to identify factors contributing to healthy trajectories (Montgomery, 2010; Vindevogel and Verelst, 2020; Popham et al., 2023).

The results of the current study indicate that both traumatic experiences and post-migration factors have a large impact on the mental health outcomes of UYRs after arrival in a host country, which is in line with previous studies of young refugee populations (Montgomery, 2010; Fazel et al., 2012; Höhne et al., 2020).

Consistent with a large body of literature (Fazel et al., 2012; El Baba and Colucci, 2018; Höhne et al., 2020), UYRs who reported more traumatic events showed higher symptom levels of PTSS, depression and anxiety. The effect remained significant even when post-migration factors were included in the analyses, highlighting the ongoing impact of traumatic events UYRs have undergone before and during flight.

The study focused on the influence of post-migration factors which represent an additional risk for UYRs’ wellbeing after controlling for the number of traumatic events. Compared to prior studies using the DSSYR questionnaire (Vervliet et al., 2014; Behrendt et al., 2022), the young participants reported a relatively high number of daily stressors (especially worries about the family, financial problems, uncertainty regarding their future, and boredom). Consistent with prior findings, the cumulation of daily stressors seems to potentiate mental distress in young refugees (Seglem et al., 2011; Vervliet et al., 2014; Keles et al., 2018b; Jensen et al., 2019; Dangmann et al., 2021; Behrendt et al., 2022). The results of the regression analyses further showed, that especially the number of social stressors (like e.g., experiences of discrimination, and boredom) predicted the severity of mental health problems, while material stressors (like e.g., lack of food or money) only had an additional impact on the prediction of symptoms of depression. Consistent with findings from a recent study (Derluyn et al., 2023), social hardships (such as problems in building friendships or being confronted with prejudice and discrimination) directly lead to distress showing that the social environment and integration is crucial for UYRs’ mental health. When basic (material) needs are met, this is indeed one of the main developmental tasks in adolescence and impacts UYRs’ wellbeing just as their peers’ wellbeing.

As one of the first studies, these analyses included an individual measure for UYRs’ feelings regarding their residential status. Regardless of the status itself or problems obtaining legal documents, individual worries about the residential status predicted higher PTSS and anxiety scores. In previous studies, high levels of distress and PTSS have also been found among UYRs with insecure residence status (Gerlach and Pietrowsky, 2012; Lamkaddem et al., 2015) and after a refusal of their asylum claim (Jakobsen et al., 2017; Müller et al., 2019; Unterhitzenberger et al., 2019).

The study further investigated the beneficial effect of post-migration factors and especially focused on sociocultural adaptation, family contact, and social support. In line with previous publications (Hollins et al., 2007; Oppedal and Idsoe, 2015; Sierau et al., 2019; Höhne et al., 2021; Behrendt et al., 2022), the findings demonstrate the positive effect of contact to family members, in the way that more frequent contact went along with less symptoms of anxiety and depression. Family members still seem to play an important role in the everyday life of UYRs. To know about the whereabouts of the family and to potentially share thoughts and problems might alleviate sadness and worries, while less contact appears to be associated with more worries and anxiety.

Practical aspects of the sociocultural adaptation to the new environment have not been the major focus of previous studies. The results of the current study show that UYRs with less problems regarding their environment (e.g., weather conditions, social and cultural norms) report lower levels of depression indicating that adaptation and integration seem to be beneficial for mental health, which is in line with findings from prior studies (Montgomery and Foldspang, 2007; Höhne et al., 2021). However, we would have expected, that a longer stay in the host country would enable a better sociocultural adaptation and better mental health outcomes along with it, but a negative effect of the duration variable on depressive symptoms has been found. This finding might be explained by growing worries of UYRs when they approach the age of adulthood and the moment when they have to leave the CYWS facility.

Contrary to prior studies (Sirin et al., 2013; Oppedal and Idsoe, 2015; Sierau et al., 2018; Popham et al., 2023), satisfaction with social support did not predict mental health problems in the current study. This might be due to methodological reasons (e.g., satisfaction with social support measured only by a single item) or due to the fact that we did not examine whether there is support from at least one adult mentor.

### 4.1. Strengths and limitations

As a strength, the study used validated and standardized self-report measures to assess mental health outcomes and post-migration stressors in UYRs which allows for comparing the study results to findings of other previous and future studies. Additionally, the study was highly heterogeneous including UYRs from different countries of origins and geographical regions and screening was performed in 22 different CYWS all over Germany. Moreover, different post-migration factors (e.g., daily stressors, sociocultural adaptation) have been considered for the analyses.

However, several limitations of the current study need to be addressed. First, our results may be influenced by selection bias, as participation in the study was voluntary and UYRs with severe or no mental health problems may have not participated in the study. Therefore, results might not be fully generalizable to the UYRs population resettled in Germany. Second, social desirability may have influenced the responses of UYRs, resulting in an underestimation of effects. Third, despite the high validity of the questionnaires for mental health outcomes, the screening instruments for PTSS, depression, and anxiety are not sufficient to obtain reliable diagnoses. In future studies, more detailed information should be obtained by using semi-structured clinical interviews to obtain symptom load and diagnoses of PTSS, depression and anxiety. Caution is also warranted when interpreting the results of the DSSYR questionnaire and the two-factor structure because a broad validation is still lacking. Fourth, the cross-sectional nature of the study does not allow for causal conclusions but provides an insight into the complex interplay of risk and protective factors for UYRs mental health. The interactions between stressors and mental health problems might also be the result of a bidirectional relationship and more complex and transactional models (Keles et al., 2016). Moreover, the correlational design precludes us from drawing conclusions about the longitudinal development of UYRs’ mental health problems, and how the impact of different risk factors varies over time. Hence, further longitudinal studies are needed to examine causal associations between pre- and post-migration stress and mental health outcomes in these populations. Fifth, with regard to the variety of geographical regions of origin, the analyses did not allow to distinguish between each different country of origin due to the small group sizes, so the results might not be comparable to studies including e.g., solely war affected participants (Popham et al., 2023). Sixth, given the high proportion of male participants (81.7%), the results cannot be easily transferred to merely female groups. However, the relatively small proportion of girls is representative for the group of UYRs in Germany (Deutscher Bundestag, 2020). Seventh, health status and medication used by UYRs was not included in the analysis due to a lack of data provided on this information. Eighth, the results have to be interpreted with caution due to the skewed distribution of the assessed variables and the small sample size, which limits generalizability of the results.

### 4.2. Conclusion and implications

The results of the study highlight both resilient and vulnerable patterns of UYRs’ mental health and emphasize the importance of traumatic experiences and post-migration factors for the mental health of UYRs after resettlement in a host country. Our study demonstrated that nearly 60% of UYRs showed alarming levels of mental health problems at least in one domaine and are potentially in high need for support to manage post-traumatic stress, daily stressors and to adapt themselves to the new environment. Even though the German residential care system is characterized by a comparably high professionalization of staff and small group sizes (Hardera et al., 2013), the examined prevalence of mental disorders is as high as in other international studies. As a consequence, there is a need for interventions that focus on both, resolving PTSS by using trauma-focused approaches, but also including strategies on how to cope with multiple daily stressors in the host country. Moreover, social workers and staff in residential care facilities play an important role in recognizing mental health problems in UYRs and helping them to get along with current stressors. They may also create technical conditions to enhance contact to family members. Additionally, policy makers and health authorities should promote an environment with reduced daily stressors, and fewer risks for new traumatization and distress. Among other measures, this comprises a long-term stability of residence and future perspectives, but also antidiscrimination campaigns and a favorable social environment.

However, the results do not allow any conclusions to be drawn about interactions between the factors and potential mediating or moderating effects. Therefore, future studies should further investigate these effects in order to gain more knowledge about conditions that have the potential to buffer the impact of negative experiences. Moreover, more data is needed on the trajectory of mental health problems of UYRs over time, especially when they leave the care system and are often left alone without intensive support. Future research should further examine differences in mental health outcomes between unaccompanied and accompanied young refugees in order to adjust actions and interventions to their individual needs.

## Data availability statement

The datasets generated for this study is available from the corresponding author on request.

## Ethics statement

The study was approved by the Ethics review board of the University Ulm (243/19) and Eichstätt-Ingolstadt (004-19). Written informed consent to participate in this study was provided by the participants and their legal guardians.

## Author contributions

RR, HK, EP, and CS contributed to the study conception and design. EP, CS, MG, JE, and FH performed the material preparation and data collection. FH and JE performed the statistical analysis and wrote the first draft of the manuscript. All authors commented on previous versions of the manuscript and read and approved the final manuscript.

## Footnotes

1 https://drks.de/search/en;registration number DRKS00017453.
